# Intellectual Capital and the Romanian organization performance in Ophthalmology

**Published:** 2020

**Authors:** Consuela Mădălina Gheorghe

**Affiliations:** * Philologist, Authorized translator

The concept of intellectual capital was first used in the last decade of the 20th century, being officially recognized by Thomas A. Stewart in 1998. 

According to Aymen Raheem Abdulaali, who wrote the paper entitled “The Impact of Intellectual Capital on Business Organization”, in the Academy of Accounting and Financial Studies Journal, “intellectual capital tends to be an important resource and a key contributor to the economic success and value creation in a business”. In other words, Intellectual capital is an intangible value trigger in an organization that should bring advantages to both the organization and its beneficiaries. This approach suggests that in terms of competitive advantage, Intellectual Capital represents an asset for any type of organization. However, literature on Intellectual Capital does not offer a commonly accepted perspective of the concept but rather provides a description of its dimensions and some examples that may be included under the Intellectual Capital umbrella. As such, according to Molodchik and Shakina (2013), the dimensions of the Intellectual Capital refer to Human Capital, Structural Capital and Relational Capital. The Human Capital encompasses the management capabilities of an organization and the knowledge and skills of the employees, whereas the Structural Capital involves innovative capabilities and internal or IT process systems. In addition, the Relational Capital consists of the networking capabilities of an organization with different stakeholders such as consumers, mass-media, suppliers, government and other partners. According to Gheorghe, Purcarea, Gheorghe (2018), health care organizations comprise a vast palette of formal and informal knowledge, both structured and unstructured, used by individuals and groups of individuals as well as revealed by intangible resources merged with embedded relationship capabilities, thus defining the Health Care Intellectual Capital. 

Barney J, who wrote the article entitled “Firm resources and sustained competitive advantage”, stated that the performance of the health care organizations and, implicitly, of the ophthalmological organization, may be explained by the different types of intellectual capital, different approaches and capacities for leveraging intellectual capital. It is a fact that Health Care Intellectual Capital cannot be totally managed to fit the aims of the ophthalmological organizations but rather improve their performance. Thus, in order to improve the performance of the Health Care Intellectual Capital, the ophthalmological organizations should focus more on training, namely on the Human Capital. Without a proper training of the Human capital, the development of the organization, the implementation of any strategy or innovation, will result into a complete failure. 

Another important issue that must be taken into consideration by the managers of the ophthalmological organizations is the volume of data that is continuously growing, being reflected in the Structural Capital. If it is not managed efficiently, the outcome may be a tremendous loss for the ophthalmological organization performance, which will also significantly influence the Human capital, leading, according to Jenna M. Evans, Adalsteinn Brown and G. Ross Baker, to “instability among leader and front-line staff, the limitations of administrative and clinical databases for supporting research and learning”. 

Moreover, the managers of the ophthalmological organizations have an important role of answering the demands of the stakeholders, thus, the Relational Capital helps identifying “the resource configurations that best support achieving the complex mandates of ophthalmological organizations”. 

In conclusion, even though it is difficult to measure, Health Care Intellectual Capital has proved to be a vital element in ensuring the survival of any ophthalmological organization, because, according to Swewart (2008), cited by Aymen Raheem Abdulaali in “The Impact of Intellectual Capital on Business Organization”, in 2018, “intellectual capital refers to skills, knowledge, experience and customer relationships that offer an organization a competitive advantage over the competitors”. Thus, it can be stated that intellectual capital is of core importance in offering a real value to any ophthalmological organization. In addition, ophthalmological organizations should use all their existent resources to ensure their success.

**Assist. Prof. Gheorghe Consuela-Mădălina, PhD,** **Philologist, Authorized translator**

